# Health-Related Quality of Life and Home Enteral Nutrition in Children with Neurological Impairment: Report from a Multicenter Survey

**DOI:** 10.3390/nu11122968

**Published:** 2019-12-05

**Authors:** Valeria Dipasquale, Marco Ventimiglia, Simone Maria Calogero Gramaglia, Barbara Parma, Caterina Funari, Angelo Selicorni, Chiara Armano, Silvia Salvatore, Claudio Romano

**Affiliations:** 1 Pediatric Gastroenterology and Cystic Fibrosis Unit, Department of Human Pathology in Adulthood and Childhood “G. Barresi”, University of Messina, 98122 Messina, Italy; dipasquale.valeria@libero.it (V.D.); simone.gramaglia10@gmail.com (S.M.C.G.); 2 Inflammatory Bowel Disease Unit, “Villa Sofia-Cervello” Hospital, 90146 Palermo, Italy; marco20miglia@gmail.com; 3 Department of Pediatrics, Sant’Anna Hospital, 22042 Como, Italy; barbaraparma79@hotmail.it (B.P.); caterinafurnari@gmail.com (C.F.); angelo.selicorni@gmail.com (A.S.); 4 Pediatric Department, “F. Del Ponte” Hospital, Insubria University, 21100 Varese, Italy; chiaraarmano@gmail.co (C.A.); silvias.varese@gmail.com (S.S.)

**Keywords:** health-related quality of life, home enteral nutrition, neurological impairment, questionnaire, caregivers, children

## Abstract

We aimed to measure the health-related quality of life (HRQoL) of children with neurological impairment (NI) on home enteral nutrition (HEN). An original survey among the caregivers of NI children on HEN for ≥ 12 months followed in three Italian centers (Messina, Como and Varese) was carried out between January 2013 and September 2018. The questionnaire was made up of 12 issues focused on the frequency with which certain situations associated with HEN are perceived, and grouped into physical and psychological/social dimensions. The results were analyzed statistically according to the age of the children, underlying disease, and the HEN programs’ duration. In total, 80 caregivers were approached, and 75 surveys were completed (an overall response rate of 93.7%). Nearly all the caregivers reported a satisfying HRQoL, ranging from acceptable (*n* = 6, 8%), to good (*n* = 22, 29.3%), to excellent (*n* = 47, 62.7%). A significant correlation was found between HRQoL and underlying NI-associated disease (*p* = 0.001). Our data showed that HEN positively affects the QoL of NI children and their caregivers. This should encourage healthcare providers to consider this nutritional intervention early in the approach to this group of patients, in order to prevent or reduce QoL impairment.

## 1. Introduction

Children with neurological impairment (NI) have feeding difficulties and gastrointestinal symptoms, usually resulting in undernutrition. The result is an overall reduced health-related quality of life (HRQoL) of patients and their caregivers. In general, quality of life (QoL) is the perceived quality of an individual’s daily life, including all emotional, social and physical aspects. In health care, HRQoL is an assessment of how the individual’s well-being may be affected by a disease, disability or disorder [1,2]. Quality of life is considered to be the most important outcome for chronic conditions such as NI [3]. It has been demonstrated that caregivers of pediatric patients with NI have a very poor QoL, worse mental health, and higher burnout levels [4]. Enteral tube feeding is increasingly being adopted, to improve the growth and nutritional status of children with NI-associated oral dysphagia and undernutrition [5]. The 2017 European Society of Pediatric Gastroenterology, Hepatology, and Nutrition (ESPGHAN) guidelines [6,7] recommend choosing enteral nutrition as a safe and good-quality nutritional intervention for NI children, since it has been shown to be effective in reversing malnutrition in this group of patients, and to positively affect family routine and social dynamics. Enteral nutrition is usually commenced in hospital, but the lifelong duration of many programs requires home enteral nutrition (HEN), thus avoiding hospitalization-related high costs and complications [8]. There is a paucity of data on whether and how HEN influences the quality of life of NI patients, especially in the pediatric group of age. The aim of this study was to evaluate the impact of HEN on the health-related QoL of a cohort of children with NI and their caregivers.

## 2. Materials and Methods 

A multicenter, cross-sectional study of NI children on HEN followed in three in 3 Italian centers (Messina, Como and Varese) was carried out. Data were collected from January 2013 to September 2018. The inclusion criteria comprised: (a) age ≤ 18 years; (b) progressive or nonprogressive NI; (c) exclusive enteral feeding; (d) ≥ 12-month duration on HEN. There were 80 potentially eligible cases. According to 2017 ESPGHAN guidelines, NI was defined as any condition associated to the central nervous system, encompassing both brain and spinal cord, with impairment of the patient’s language, cognitive, and motor skills. This study was conducted in conformity with the principles and regulations of the Helsinki Declaration. Informed consent was obtained from the children’s parents/legal guardians, who were fully informed of the nature and purpose of the study. The study protocol was approved by the Ethical Institutional Review Board of Institutional Ethics Committee of Messina University Hospital.

### 2.1. QoL Evaluation

The instrument used to measure QoL in NI children was an original survey made up of 12 issues of HEN-related QoL, focusing on the frequency with which certain situations associated to HEN are perceived. All issues were grouped into physical and psychological/social functioning. Physical functioning issues assessed the easiness of feeding the child, physical aspects, weight, physical discomfort, respiratory symptoms, and HEN preparations management. The psychological/social dimensions investigated overall psychological and physical aspects, daily tasks, mealtimes, sleep patterns, and the ability to go out with friends. The score for each issue was 0 (no, never), 1 (yes, sometimes), and 2 (yes, always). The total score was given by the sum of the 12 items and ranged from 0 (the worst HEN-related quality of life) up to 24 (the best HEN-related quality of life) (Table 1). 

The results were analyzed statistically according to the age of the children, underlying disease, and the HEN programs’ duration. 

Primary caregivers were involved to answer on behalf of patients, who were not able to answer the questionnaire by themselves due to the NI. Caregivers had to be adults (≥18 years) making, at most, 3 or 4 errors in the Short Portable Mental Status Questionnaire [9], hereon referred to as the Pfeiffer’s test.

### 2.2. Data Collection

The questionnaire was administered by telephone interview. Variables including age, sex, underlying NI-associated disease, and the modality of HEN were extracted from patients’ medical records. 

### 2.3. Statistical Analysis

Continuous variables were reported as medians with interquartile ranges [IQR], and categorical variables as frequency and percentage. Fisher’s exact test was used to assess dependence between categorical variables; Spearman’s rank correlation coefficient (ρ) was used to measure correlation between continuous variables; two-tailed tests were performed to assess statistical significance. Results were considered statistically significant when *p* ≤ 0.05.

Multiple Poisson Generalized Linear Model (GLM) was used to determine the association between the QoL Score and possible predictors.

## 3. Results

### 3.1. Caregivers Demographics

Of the caregivers who completed the questionnaire, 65% (*n* = 48) were the child’s mother, 29.3% (*n* = 22) were the child’s mother and father, 5.3% (*n* = 4) the child’s grandmother, and 1.3% (*n* = 1) the child’s father. The median age of the mothers was 29 years (range: 20–55 years), and for fathers it was 33 years (range: 22–50 years). The mean Pfeiffer’s test score was 0.5. 

### 3.2. Children Demographics and Clinical Variables

In all 80 NI pediatric patients on HEN, 93.75% (*n* = 75) participated in the study. The children’s main demographic and clinical characteristics are summarized in Table 2. 

The median age was 9 years (range: 5–12.9 years), and 54.6% (*n* = 41) were male. Most of the patients (58.7%, *n* = 44) suffered from a neurological disease, mainly cerebral palsy. Other diagnoses included genetic (34.6%, *n* = 26) or metabolic (6.7%, *n* = 5) disorders. All the children in this study were classified using the Gross Motor Function Classification Scale [10] as either level IV or V (severe motor disability). Percutaneous endoscopic gastrostomy was the most commonly used type of enteral access (*n* = 70, 93.3%), in line with ESPGHAN recommendations for long-term (>2 months) nutritional intervention. Only 3 children had gastro-jejunostomy or jejunostomy because of poor tolerance to gastrostomy. No child was fed by mouth or naso-gastric tube, or a combination of both. All children received a standard energy density (1 kcal/mL) of polymeric or semi-elemental formula. Enteral tube feeding was administered as boluses over a 24 h period in most of the children, while a combination of overnight continuous feeds with boluses during the day was chosen for six patients because of their poor tolerance to volume. The mean age at HEN beginning was 2 years (range: 0.9–6 years), and the mean HEN duration was 4 years (range: 2–8 years). 

### 3.3. Caregivers Interviews

In total, 80 caregivers were approached, and 75 surveys were completed for an overall response rate of 93.75%. Table 3 contains summary responses to each of the 12 questionnaire issues. 

**Figure 1:**
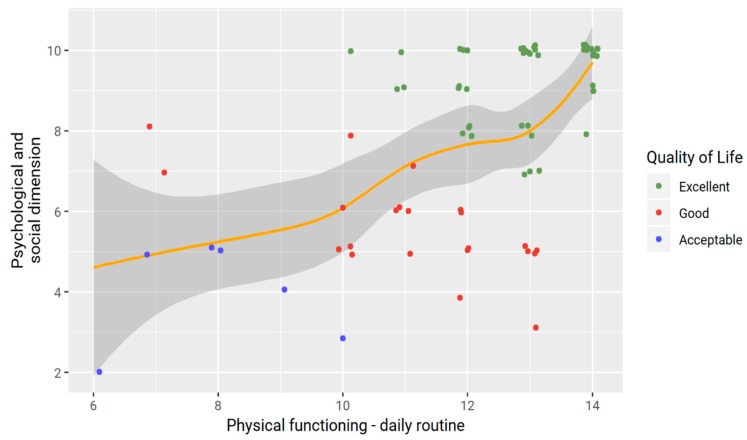
Correlation between physical functioning (daily routine) and psychological and social dimensions.

With regard to physical functioning, nearly all of the caregivers (64 out of 75) stated that HEN always makes feeding the child easier. A perceived improvement of the physical aspects and weight of the children and caregivers was reported by 85.3% (*n* = 64) and 86.7% (*n* = 65), respectively. A relief or disappearance of respiratory symptoms was reported by 34 (always) and 30 (sometimes) out of 75 caregivers. HEN preparations were considered easier to obtain (*n* = 52, 69.3% always) and use (*n* = 73, 97.3%). Similarly, an improvement in both the psychological and social dynamics was reported, since most of the caregivers reported to be able to go out with friends (*n* = 40, 53.3% always, *n* = 24, 32% sometimes), sleep well at night (*n* = 44, 58.7% always), and carry out routine activities (*n* = 51, 68% always). A significant positive correlation was found between physical, psychological and social functioning (ρ = 0.58, *p* < 0.001) (Figure 1). Based on total scores, nearly all of the caregivers of NI children receiving HEN reported a satisfying HEN-related QoL (acceptable, *n* = 6, 8%; good, *n* = 22, 29.3%; excellent, *n* = 47, 62.7%). 

**Figure 2:**
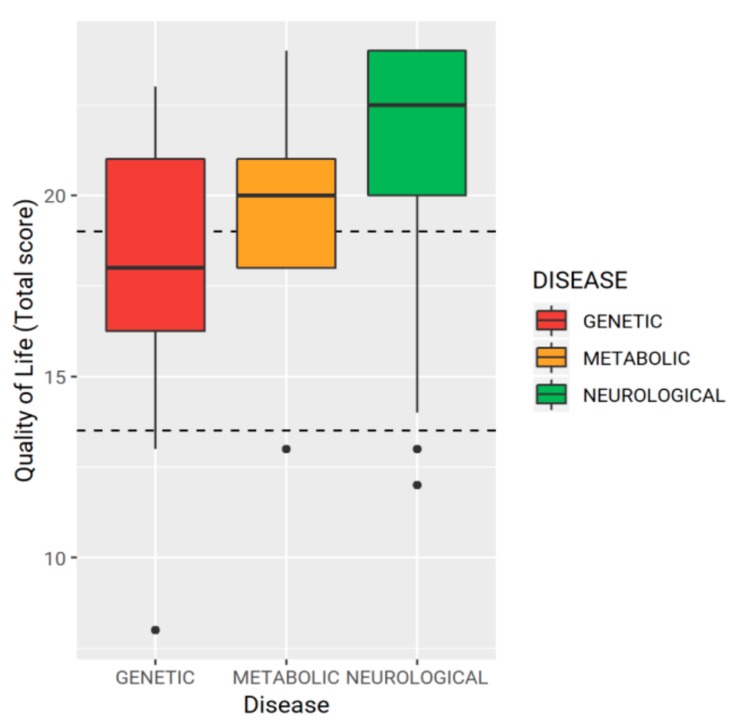
Underlying neurological impairment-associated disease and total scores for quality of life.

A statistically significant correlation was found between HRQoL and underlying NI-associated disease. Children with neurological disorders (cerebral palsy) had higher total HRQoL scores than those with genetic or metabolic diseases (excellent HRQoL in 79.5% vs. 34.6% patients, respectively, *p* = 0.001) (Figure 2). The age of the children at HEN beginning and the HEN programs’ duration showed no significant correlation with the HRQoL (*p* = 0.31 and *p* = 0.47, respectively). A multiple regression analysis regarding the HRQoL of NI children on HEN was also performed, and confirmed that children with cerebral palsy had a better HRQoL than children with genetic or metabolic diseases (β: 2.65, 95% CI 0.87–4.41, *p* = 0.005). Notably, a marginally significant negative correlation was found between HRQoL and the HEN programs’ duration (β: −0.19, 95% CI −0.40–0.03, *p* = 0.098), as the HRQoL score seems to decrease by 0.19 points for each year spent on HEN. Multiple regression models did not show further significant factors. 

## 4. Discussion

Neurological disability is one of the first indications of HEN in childhood, and usually begins early in life [11,12]. A 14-year multicenter study, carried out in four Italian centers between 1996 and 2009, found 757 recorded cases of HEN, of which almost half were supporting NI children with inadequate oral intake and chronic undernutrition [12]. As in our cohort of patients, the median age at HEN beginning was 2 years. The median duration was 8.1 months, and the longest programs (between 25 months and 175 months) were those supporting NI children. More recently, a median HEN duration of 6 years has been reported [13]. Although the use of HEN has progressively increased in many countries [11,12,14,15], data on the impact of HEN on the QoL of NI children and their caregivers are scarce. Previous qualitative studies have reported social isolation, and difficulties in obtaining care among parents of children with NI who are fed through a gastrostomy tube [16,17,18,19]. Using a validated instrument for measurement of QoL, Sullivan et al. [20] found a significant improvement in the QoL of caregivers, both at 6 and 12 months after starting a gastrostomy tube feeding in children with cerebral palsy. Caregivers (the child’s mother in more than 90% of cases) reported significant improvements in social functioning, mental health, vitality, and in general health perception after starting enteral tube feeding. Similarly, Grzybowska-Chlebowczyk et al. [21] reported that the placement of a PEG tube improved the lives of more than 70% of the caregivers of 302 children. Unfortunately, HEN-related QoL was not explored. Children with severe NI are not able to self-report their perceptions of their HRQoL, therefore parent-proxy reports are the only available measures [3]. In our survey, all data on HRQoL have been obtained from interviews with caregivers. Caregivers reported a marked improvement of their HRQoL, in both physical and psychological/social dimensions. The easier feeding practice (“it’s easier to feed the child”) led to a significant improvement in eating and feeding time, which, together with a perceived reduction in physical discomfort related to feeding (coughing, gagging, etc.) and better physical aspects, could lead to reduced concerns about the child’s nutritional status. Moreover, caregivers reported a greater ease in performing daily activities, and better psychological and social functioning, even if percentages for issues belonging to social/psychological dimensions were slightly lower than physical ones, probably because a child with severe NI per se requires close and ongoing support, regardless of feeding practice. To our knowledge, this is the first pediatric study finding a significant negative correlation between underlying NI-associated disease and HEN-related QoL. A favorable influence of the nonprogressive nature of neurological impairment due to cerebral palsy could be hypothesized, but further studies are needed. The marginally statistical association between the HEN programs’ duration and HEN-related QoL could be explained by the occurrence of tube-related complications, which were not evaluated in our study. However, tube-related complications are frequent in children receiving long-term HEN [6,20,22]. Even if these complications are not life threatening, they are associated with increased healthcare utilization [22]. Our finding of no association between HEN-related QoL and the age of the children is in agreement with a previous pediatric study [21].

Any conclusions drawn from the findings of this research study must be qualified in light of the study’s limitations. Firstly, it is a retrospective study, so recruited cases and clinical management are the confounding variables. Moreover, being a retrospective study, this may be confined only to our clinical findings and management. Secondly, the health-related QoL of children with NI and their caregivers before starting enteral tube feeding has not been evaluated. Nonetheless, current literature data, including consensus statements and international guidelines, clearly state that NI children have a reduced health-related QoL, due to feeding difficulties and gastrointestinal symptoms. Thirdly, regarding metabolic and genetic diseases, we did not differentiate between different nosological entities. A multiple regression analysis was not made in relation to single gastrointestinal symptoms (e.g., constipation, abdominal pain), or complications related to gastrostomy placement. The strength of this study is that the indications of nutritional intervention are in conformity with current evidence-based recommendations. 

## 5. Conclusions

In conclusion, this survey investigated the impact of HEN on the HRQoL of pediatric patients with NI and their caregivers. The main finding was that HEN positively affects the QoL of NI children and their caregivers. This should encourage healthcare providers to consider this nutritional intervention early in the approach to this group of patients, in order to prevent or reduce QoL impairment. Caring for a child with NI is a major challenge for general pediatricians and families. Research revolving around HRQoL is crucial, because of the implications that it can have on current and future treatments. 

## Figures and Tables

**Table 1:** Total scores of HEN-related quality of life.

Total Score	HEN-Related Quality of Life
0–6	Poor
7–13	Acceptable
14–19	Good
20–24	Excellent

HEN, Home enteral nutrition.

**Table 2:** Patients’ demographic and clinical variables.

Variable	Total (*N* = 75)
Male, *N* (%)	41 (54.6)
Current age, years, median (IQR)	9 (5, 12.9)
Main disease, *N* (%)	
Neurological	44 (58.7)
Genetic	26 (34.6)
Metabolic	5 (6.7)
Administration route, *N* (%)	
Gastrostomy	70 (93.3)
Gastro-jejunostomy	2 (2.6)
Jejunostomy	1 (1.3)
Age at HEN beginning, *N* (%)	
Median, (IQR)	2 (0.9, 6)
<6 years	54 (72)
6–10 years	10 (13.3)
>10 years	11 (14.7)
HEN duration, years, median, (IQR)	4 (2,8)

HEN, Home enteral nutrition; IQR, inter-quartile range.

**Table 3:** Caregivers’ response to questionnaire items.

Items		Response, *N* (%)		
		Yes Always	Yes Sometimes	No Never
**Physical functioning and daily routine**	It’s easier to feed the child	64 (85.3)	9 (12)	2 (2.7)
	I perceive that the physical aspect of the child is improved	64 (85.3)	10 (13.4)	1 (1.3)
	I perceive that the weight of the child is gained	65 (86.7)	8 (10.6)	2 (2.7)
	I perceive that with HEN physical discomfort related to feeding is reduced or disappeared	48 (64)	24 (32)	3 (4)
	I perceive that the respiratory symptoms are reduced or disappeared	34 (45.3)	30 (40)	11 (14.7)
	I perceive that HEN preparations are easier to get (for instance, they are available in pharmacies, it is easy for me to get a prescription)	52 (69.3)	18 (24)	5 (6.7)
	I perceive that HEN preparations are easier to use	73 (97.3)	2 (2.7)	0
**Psychological and social dimension**	I can maintain the usual meal times	56 (74.7)	14 (18.6)	5 (6.7)
	I can continue doing my daily tasks (read newspapers, cook, work etc)	51 (68.0)	21 (28)	3 (4)
	I can sleep well during the night	44 (58.7)	24 (32)	7 (9.3)
	I can go out with my friends	40 (53.3)	24 (32)	11 (14.7)
	I perceive that my psychological and physical aspect are improved	51 (68)	17 (22.7)	7 (9.3)

HEN, Home enteral nutrition.
